# The role of m5C methyltransferases in cardiovascular diseases

**DOI:** 10.3389/fcvm.2023.1225014

**Published:** 2023-07-05

**Authors:** Yan-Yue Wang, Yuan Tian, Yong-Zhen Li, Yi-Fan Liu, Yu-Yan Zhao, Lin-Hui Chen, Chi Zhang

**Affiliations:** ^1^ Key Lab for Arteriosclerology of Hunan Province, Institute of Cardiovascular Disease, Hengyang Medical School, University of South China, Hengyang, China; ^2^ Research Laboratory of Translational Medicine, Hengyang Medical School, University of South China, Hengyang, China

**Keywords:** RNA modification, m5C methyltransferases, cardiovascular diseases, atherosclerosis, mitochondrial dysfunction

## Abstract

The global leading cause of death is cardiovascular disease (CVD). Although advances in prevention and treatment have been made, the role of RNA epigenetics in CVD is not fully understood. Studies have found that RNA modifications regulate gene expression in mammalian cells, and m5C (5-methylcytosine) is a recently discovered RNA modification that plays a role in gene regulation. As a result of these developments, there has been renewed interest in elucidating the nature and function of RNA “epitranscriptomic” modifications. Recent studies on m5C RNA methylomes, their functions, and the proteins that initiate, translate and manipulate this modification are discussed in this review. This review improves the understanding of m5C modifications and their properties, functions, and implications in cardiac pathologies, including cardiomyopathy, heart failure, and atherosclerosis.

## 1. Introduction

Cardiovascular disease (CVD) is the leading cause of death in many parts of the world, with an estimated 18 million deaths each year (1). By 2030, more than 23.3 million people will die from CVD, according to the World Health Organization (WHO) (2, 3). As the world’s leading cause of death, we must better understand the cellular, molecular, and genetic origins of CVD (4). There are currently a number of clinical trials investigating the safety and efficacy of RNA therapeutics in clinical conditions such as CVD (5). Over a hundred chemical modifications can be made to cellular RNA, and these modifications have been recently found to be important for posttranscriptional regulation (6–8). Most posttranscriptional modifications of RNA are conserved throughout evolution and can be found in all kingdoms of life (9). RNA methylation, which is the most prevalent epigenetic modification of RNA nucleotides, with over 170 different modifications reported thus far, primarily manifests as 7-methylguanosine (m7G), 5-methylcytosine (m5C) (Table 1), 5-hydroxymethylcytosine (5-hmC), N1-methyladenosine (m1A), N6-methyladenosine (m6A), N6, 2′-O-dimethyladenosine (m6Am), and 2′-O-methylation (2′-OMe) (10). M5C has been found to be highly abundant in eukaryotic mRNA molecules. Transcriptome analysis has identified more than 10,000 m5C sites (11). Archaea has also been reported to contain this modification, but it has not yet been identified in bacterial mRNA molecules (12). There is growing evidence that m5C RNA modifications play a broad role in RNA metabolism, including RNA export, the stability, efficiency, accuracy of translation, and long-distance RNA transport (13). The focus of this review article will be exploring the effect of m5C methyltransferases on CVD pathology since this modification, although there are a great number of other modifications, has been shown to be pivotal in CVD.

**Table 1:** A list of abbreviations.

Non-standard Abbreviations and Acronyms	
m5C	5-methylcytosine
NSUN	NOL1/NOP2/SUN domain
DNMT	DNA methyltransferase
TRDMT	tRNA-specific methyltransferase
TET	Ten-eleven translocator family
ALKBH1	f5C by alkb homolog 1
ALYREF	Aly/REF export factor
YBX1	Box-binding protein 1
Pol II	Polymerase II
HHcy	hyperhomocysteinemia
elF2*α*	eukaryotic initiation factor 2 alpha
sncRNAs	small noncoding RNAs

## 2. 5-methylcytosine

**Figure 1:**
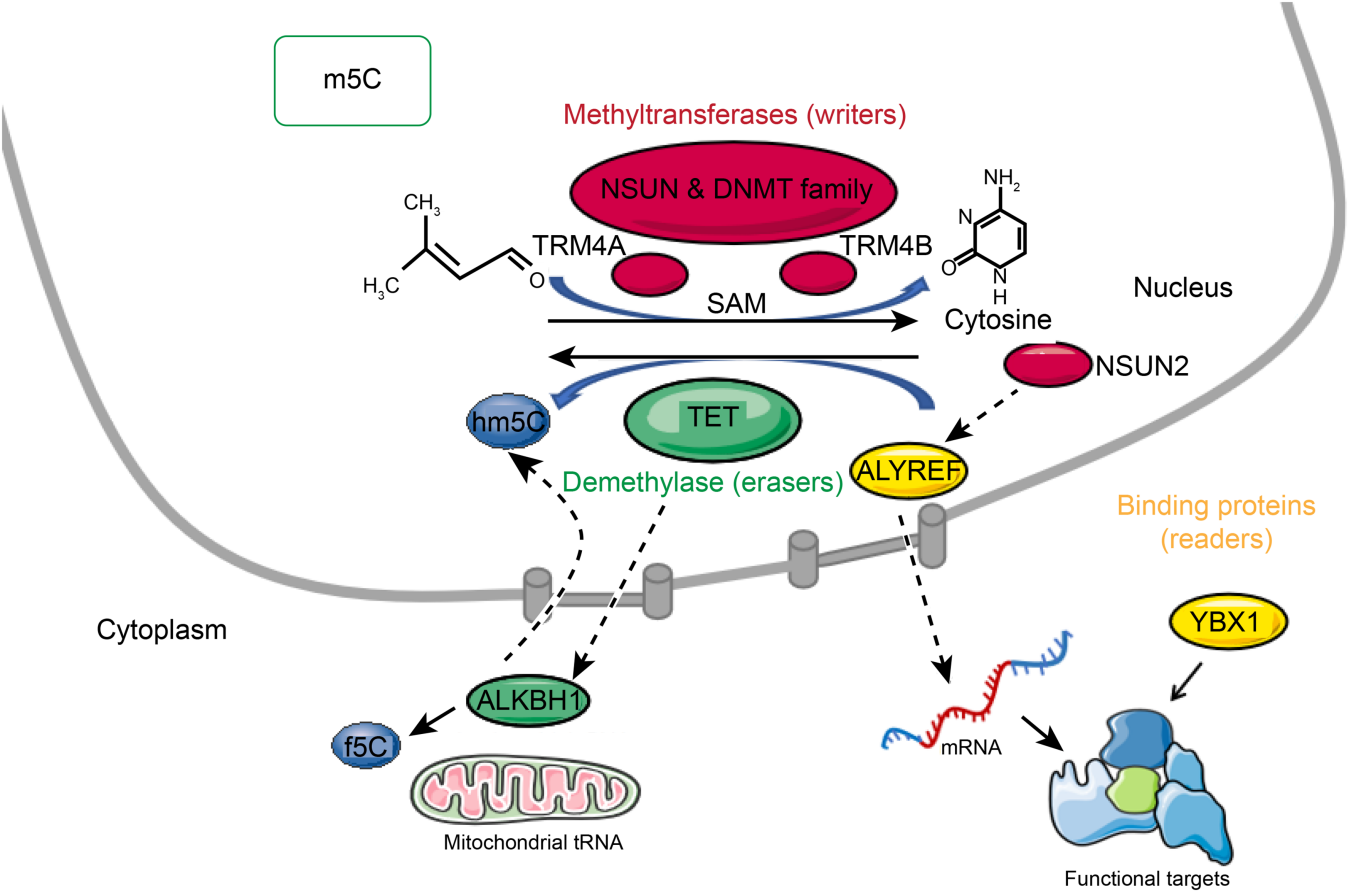
Reversible m5C mRNA modification. A methyltransferase (writer) and a demethylase (eraser) can reversibly regulate the methylation levels of m5C. The majority of m5C molecular functions are accomplished by binding proteins (readers). M5C is formed by methyltransferases that use S-adenosylmethionine (SAM) as the methyl donor and transfer the methyl group to cytosine. M5C methyltransferases include NSUN family members, DNMT, and TRDMT families. M5C can be oxidized by TET enzymes in mRNA to produce 5-hydroxymethylcytosine (hm5C), and f5C by alkb homolog 1 (ALKBH1) is formed at the mitochondrial tRNA wobble position. The binding partner ALYREF and the methyltransferase NSUN2 of m5C regulate mRNA export. YBX1 is a cytoplasmic mRNA m5C reader protein.

A methyltransferase (writer) and a demethylase (eraser) can reversibly regulate the methylation levels of m5C. The majority of m5C molecular functions are accomplished by binding proteins (readers). Recent studies have demonstrated that m5C plays multiple molecular roles in RNA processing, including mRNA export, RNA stability, translation and long-distance RNA transport (Figure 1) (13).

### 2.1. M5C writers

M5C is formed by methyltransferases that use S-adenosylmethionine (SAM) as the methyl donor and transfer the methyl group to cytosine (14). As many as 10 RNA m5C methyltransferases are known, including NOL1/NOP2/SUN domain (NSUN) family members, DNA methyltransferase (DNMT) homologs, and tRNA-specific methyltransferase (TRDMT) families. The NSUN family includes several variants (NSUN1 to NSUN7) and NSUN5a/b/c (15–17). In *Arabidopsis thaliana*, TRM4A and TRM4B are members of the TRDMT family (18, 19). Different cysteine residues are used in the catalysis of m5C RNA and m5C DNA methyltransferases. Enzymes of the NSUN and DNMT families contain amino acid motifs IV and VI. In the NSUN family, cysteine at position VI disrupts carbon 6 of the target cytosine by nucleophilic interaction (14, 20). As a result of hydrogen interactions with the proline and aspartate sidechains in motif IV, the nucleobase is oriented in an active position, and transient protonation is promoted. The methyl group from the donor SAM is then recognized by the activated base (14).

### 2.2. M5C erasers

The role of m5C writers is now well-documented, but the role of m5C erasers is still controversial. It has been reported that m5C can be oxidized by enzymes of the ten-eleven translocator family (TET) in mRNA to produce 5-hydroxymethylcytosine (hm5C) (21, 22), and f5C by alkb homolog 1 (ALKBH1) is formed at the mitochondrial tRNA wobble position. Although f5C deposition in mitochondrial tRNAs has been established biologically, hm5C deposition in mRNAs remains unclear in terms of its biological relevance (23–25). In DNA, the TET (ten-eleven translocation) family proteins, including TET1, TET2, and TET3, catalyze 5mC to 5-hydroxymethylcytosine (5hmC). It has been shown that TETs are also RNA demethylases. Overexpression of TETs can significantly increase the RNA level of 5hmC (21). Moreover, TET1 mediates the oxidation of 5-formylcytosine (f5C) into 5-carboxycytosine (5caC) in RNA, and TET2 regulates the oxidation of m5C in mRNA, preventing 5-methylcytosine from forming double-stranded RNA. TET2-catalyzed RNA hm5C also involves RNA degradation, suggesting that it plays an important role in posttranscriptional regulation (26). ALKBH1 was identified as a mitochondrial DNA and RNA dioxygenase (27). On mt-tRNA^Met^ and the anticodon of cytoplasmic tRNA^Leu^, ALKBH1 catalyzes the conversion of m5C34 to hm5Cm34 (5-hydroxymethyl-2′-O-methylcytidine) and f5Cm34 (5-formyl-2′-O-methylcytidine). There was a significant reduction in mitochondrial translation and oxygen consumption upon deletion of ALKBH1, suggesting that ALKBH1-mediated RNA m5C metabolism might play an important role in mitochondrial function. It is also interesting to note that ALKBH1 is also involved in demethylating N1-methyladenosine (m1A) within cytoplasmic tRNAs (24, 28). ALKBH1 can also specifically act on histone H2A as a histone dioxygenase, in addition to different DNA or RNA chemical modifications (29, 30).

### 2.3. M5C readers

Most of the biological functions of RNA modifications are related to the proteins that bind to them. Recent studies have demonstrated the ability to pull down m5C-modified oligos by affinity chromatography-mass spectrometry in the mRNA of multiple species and have shown that 5-methylcytosine mediates the nuclear export of mRNAs. Knockdown of Aly/REF export factor (ALYREF), an mRNA transport adaptor, did not affect the nuclear export of nonm5C-bearing mRNA. Therefore, ALYREF seems to be a true m5C “reader” protein that regulates mRNA fate according to its m5C status (31). ALYREF has been reported to be involved in nuclear-cytoplasmic shuttling and is enriched in nuclear speckles containing pre-mRNA processing factors. ALYREF protein is retained in the nuclear compartment when NSUN2 levels are depleted, but its total cellular level remains the same. The binding partner ALYREF and the methyltransferase NSUN2 of m5C regulate mRNA export. In contrast to ALYREF, YBX1 (Y-box-binding protein 1) is a cytoplasmic mRNA m5C reader protein. YBX1 recognizes m5C through the indole ring of W65 in its cold-shock domain (32). Different tissues or species express different reader proteins, indicating that m5C modifications are functionally specific. In summary, it is necessary for the whole dynamic modification system mediated by m5C to be clarified along with the role of different components in m5C-mediated RNA metabolism, including the role of methyltransferases, demethyltransferases, and reader proteins, to help improve the understanding of RNA processing.

## 3. Cellular functions of m5C RNA methyltransferases and their catalytic mechanisms

### 3.1. NSUN1

NSUN1, also known as p120, NOL1, and NOP2 (S. cerevisiae), directly binds to 60–80S preribosomal particles and catalyzes C4447 to m5C in human 28S rRNA and C2870 to m5C in yeast 25S rRNA (33). NSUN1 expression enhances the proliferation capacity of human cancer cells regardless of their histological origin. NSUN1 can participate in gene expression regulation through bromodomain-containing protein 4 (BRD4) and Pol II recruitment in 5-AZA-resistant leukemia cell lines (34–36). According to a recent study, this protein participates in the m5C methylation of the HIV TAR RNA (trans-activating response element) by and competing with the HIV-1 Tat protein for TAR binding (37).

### 3.2. NSUN2

There is another RNA m5C methyltransferase present in eukaryotes, NSUN2 (NOP2/SUN family, member 2) (14, 38–41). Researchers have found that NSUN2, which catalyzes 5mC methylation in tRNAs C48/49/50, contributes to the stability of tRNAs and protein synthesis by mediating methylation in the variable loop of tRNA (42). In contrast, m5C mediated by NSUN2 is widespread within the coding sequence (CDS) in mRNA. According to several studies, knockdown of NSUN2 significantly decreased the methylation levels of almost 40% (2,016 of 5,063) of m5C sites from 1,144 mRNAs. It was found that 92% (577 out of 629) of the m5C sites from 500 mRNAs were NSUN2-dependent in HeLa cells with NSUN2 knockout (43).

### 3.3. NSUN3

Over a decade ago, NSUN3 was found to belong to the NSUN (Nol1/Nop2/Sun domain) family of m5C RNA methyltransferases (44). In human cells, NSUN3 is localized to the mitochondrial matrix and is the last uncharacterized member of the m5C methyltransferase family. NSUN3 specifically interacts with mitochondrial tRNA^Met^, and is responsible for introducing a m5C modification at the swing position. NSUN family m5C methyltransferases replace the cysteine in motif IV of the TCT tripeptide with alanine (C265A). A conserved catalytic cysteine requirement and the efficient cross-linking of NSUN3 to 5-azacytidine (5-AzaC)-containing mitochondrial (mt-)tRNA^Met^ strongly support NSUN3’s role as an active m5C RNA methyltransferase. NSUN3 uses the conserved mechanism of the NSUN family to mediate the m5C methylation of mitochondrial tRNA^met^ (24).

### 3.4. NSUN4

In previous studies, NSUN4 has been reported as a m5C-methyltransferase involved in methylating an unknown 16S rRNA residue (45–47). A subsequent study demonstrated that NSUN4 is capable of independently methylating C911 in human mitochondrial 12S rRNA (m5C911) (48). NSUN4 forms a stable complex with MTERF4 (methyltransferase-like 14) on the large subunit of the ribosome and is crucial to mitochondrial ribosome assembly and translation (45, 46).

### 3.5. NSUN5

It has been reported that NSUN5 catalyzes both m5C3782 and m5C3438 in human and mouse 28S rRNA, respectively (49). A nonmethylated C3782 position in 28S rRNA may inhibit global protein synthesis, promoting a translational stress response in gliomas (50).

### 3.6. NSUN6

PUA domains are RNA-binding domains found in many proteins, including YebU, YccW, RlmO, and m5C methyltransferases (51–55). PUA domains recognize the D-stem regions and CCA ends of tRNA, whereas methyltransferase domains recognize the base and ambient residues precisely. It has been shown that the CCA tail can be accurately identified by the PUA domain of NSUN6, then a precise recognition of the target site C72 by the NSUN6 catalytic core occurs when U73 binds to the NSUN6 RNA-recognition motif (RRM). It is important for tRNA recognition to have the second and third base pairs (2:71 and 3:70) of the acceptor stem. Lys248, Asp323, Cys326, and Cys373 are strictly conserved at the active site of NSUN6 in the RNA m5C methyltransferase (56). Additionally, NSUN6 interacts with two specific base pairs of the D-stem in the tRNA substrate (11:24 and 12:23) (57). *Pyrococcus horikoshii* OT3 (*P. horikoshii*) contains five genes that encode RNA m5C methyltransferases: PH1537, PH1374, PH1078, PH1991, and PH0851. It has been demonstrated that OT3 and PH1991 of *P. horikoshii* are homologs of human NSUN6 and are capable of catalyzing the modification of m5C72 along with tRNA. M5C72-modified tRNAs were slightly more thermally stable than nonmodified tRNAs (58).

### 3.7. NSUN7

Enhancer RNAs (eRNAs), which are transcriptionally regulated by enhancers in a tissue-specific manner, are a recent addition to the list of regulatory noncoding RNAS (ncRNAs) (59). Enhancer RNAs are activated by NSUN7 by promoting m5C deposition coupled to coactivator PGC-1α. PGC-1α is important for adaptive metabolic responses, suggesting that eRNA m5C modification by NSUN7 regulates metabolism (60).

### 3.8. DNMT

Although Dnmt2 possesses motifs typical of DNA methyltransferases rather than RNA methyltransferases, it has been shown to methylate RNA. The enzyme also acts as a tRNA methyltransferase and is responsible for m5C38 in tRNA^Asp^ (14, 61).

## 4. Pathophysiological mechanism of m5C RNA modifications in cardiovascular disease

### 4.1. Atherosclerosis

#### 4.1.1. Inflammation

Atherosclerosis begins with vascular inflammation, which is a pivotal event (62). Endothelial and leukocytic adhesion molecules and their shedding are essential for mediating interactions between endothelial cells and blood components or extracellular matrix (63, 64). Intercellular adhesion molecule-1 (ICAM-1) plays a crucial role in inflammation and immune responses as an immunoglobulin-like protein found in leukocytes and endothelial cells (65). Adhesion molecules on the surface of circulating mononuclear cells mediate their attachment to vessel walls during atherosclerosis, including vascular cell adhesion molecule 1 (VCAM-1), platelet cell adhesion molecule 1 (PECAM-1), and ICAM-1. It was found that in apoE-deficient mice, gene deletion of ICAM-1 reduced monocyte recruitment, which resulted in protection from atherosclerotic lesions (66–68). Methylation of RNA is a critical modification that occurs extensively in ncRNA (10–12) and mRNAs (13, 14). The tRNA methyltransferase NSUN2 promotes cell proliferation in response to Myc activation. Recent studies have shown that homocysteine (Hcy)-induced NSUN2 methylates ICAM-1 mRNA and promotes ICAM-1 translation. NSUN2 plays a role in vascular inflammation and allograft avascular necrosis by increasing ICAM-1 levels (Table 2) (69). Atherogenesis induced by hyperhomocysteinemia (HHcy) is associated with the upregulation of IL-17A expression in T lymphocytes (70); however, the mechanism underlying this process remains unknown. Hcy is an intermediate product of the methionine cycle, which produces methyl donors (8). HHcy is a condition with elevated plasma Hcy levels, resulting in chronic inflammation (71, 72). It has been reported that IL-17A mRNA is methylated in the coding region (CR) by NSUN2. NSUN2 promotes the translational expression of IL-17A, which may be responsible for HHcy-induced activation of T lymphocytes. There is evidence that NSUN2-mediated RNA methylation may have a broader impact on aging-related declines and pathologies. The interest in understanding the impact of RNA modifications has increased, and studies have shown that RNA-modifying enzymes can regulate gene expression programs by influencing the levels of proliferative proteins through the RNA-methyltransferase NSUN2 (Table 2) (73). Additionally, Hcy-induced metabolic disorders have been shown to contribute to the development of CVDs through DNA methylation (12).

**Table 2:** The association between m5C methylation and CVDs.

CVD	m5C-related molecules	Expression	m5C levels	Main functions	References
Atherosclerosis	NSUN2	Upregulated	Increased	By increasing ICAM- 1 translation, NSUN2 increased leukocyte adhesion to the endothelium.	(69)
				HHcy-induced NSUN2 methylation affected the translational level of IL- 17A, which may play a role in activating T lymphocytes.	(73)
	ALKBH1	Downregulated	Reduced	In ALKBH1-knockout cells, mitochondrial translation and oxygen consumption were strongly reduced.	(28)
	NSUN3	Downregulated	Reduced	A4435G and C4437U in mt-tRNAMet prevented NSUN3 from forming m5C34.	(25)
	DNMT2	Upregulated	Increased	DNMT2 overexpression protected substrate tRNAs from angiogenin degradation.	(97)
Heart failure	DNMT2	Downregulated	Reduced	In DNMT2 knockout cells, lncRNA RN7SK was disintegrated from the p-TEFB complex, triggering RNA pol II C-terminal domain phosphorylation, enhancing hypertrophy gene transcription.	(101)
Cardiomyopathy	NSUN4	Downregulated	Reduced	NSUN4 improved the m5C modification at C911 residues, which regulates mitochondrial ribosome assembly and mitochondrial gene translation.	(48)

#### 4.1.2. Mitochondrial dysfunction

Altered mitochondrial function is currently recognized as an important factor in the initiation and progression of atherosclerosis. The barrier and metabolic functions of artery wall cells make them particularly vulnerable to mitochondrial dysfunction. Atherosclerosis occurs when mitochondria alter metabolic and respiratory processes and produce excessive quantities of reactive oxygen species (ROS), leading to oxidative stress. These processes contribute to vascular disease and chronic inflammation associated with atherosclerosis (74). Studies have shown that ALKBH1 is responsible for the biogenesis of 5-hydroxymethyl-2’-O-methylcytidine (hm5Cm)34 and f5Cm34 in cytoplasmic (ct)-tRNA^Leu^ (CAA) as well as f5C34 in mitochondrial (mt)-tRNA^Met^. In ALKBH1-knockout cells, mitochondrial translation and oxygen consumption were strongly reduced, indicating that ALKBH1 is required for mitochondrial function (Table 2) (28). Mitochondrial gene expression dysfunction caused by mutations in mitochondrial or nuclear genomes can also play a crucial role in human diseases, including atherosclerosis and CVD (75). Two pathogenic mutations (A4435G and C4437U) in mt-tRNA^Met^ prevent NSUN3 from forming m5C34 (Table 2) (25). Mitochondrial diseases caused by these mutations include MIEH (maternally inherited essential hypertension) (76, 77). As a result of hypertension, atherosclerosis is more likely to develop (78). An increasing number of mutations have been identified in nuclear genes that shape mtRNA epitranscriptomes (23, 79–86). In the pathogenesis of atherosclerosis, genetic factors play a crucial role; however, the precise mechanisms are still not fully understood. Even so, family history analysis and gene variant screening are effective strategies to predict the development of atherosclerosis in individuals who are susceptible to hypertension (87).

#### 4.1.3. Stress reaction

Stress is a fundamental concept in biology that has been extensively applied in various fields, including psychology, physiology, social sciences, and environmental studies. As the concept of homeostasis becomes more defined, the concept of stress is also becoming more specific. For example, oxidative stress specifically refers to a disturbance in redox signaling and regulation.Endoplasmic reticulum stress is caused by the buildup of unfolded proteins in the endoplasmic reticulum, leading to cellular stress and dysfunction (88). The Integrated Stress Response (ISR) is a signaling network that is conserved throughout evolution and operates within cells to help them adapt to changing environmental conditions. Its role is to maintain the health of cells, tissues and organisms by enabling them to respond effectively to various stressors (89). Human diseases may be caused by aberrant posttranscriptional methylation of RNA. Radical reprogramming of protein translation is required by mammalian cells exposed to adverse environmental conditions (90). An integrated stress response (ISR) can be activated when eukaryotic cells are stimulated by diverse stimuli by phosphorylating eukaryotic initiation factor 2 alpha (eIF2a). Atherosclerosis in mice and humans shows persistent phosphorylation of eIF2a, a key factor in translation control (88). Angiopoietins (Angs) regulate angiogenesis. These proteins are known to promote the development of atherosclerosis (91). Angiogenin is a secreted ribonuclease, and angiogenin activates a stress-response program in mammalian cells by cleaving tRNA. The cleavage of tRNAs in eukaryotes is a conserved response to several stress stimuli (92–95). Researchers have found that angiopoietin and stress-induced tRNA cleavage are newly discovered components of mammalian stress responses. Moreover, activation of phospho-eIF2-independent translation was promoted by transfection of angiogenin-induced tRNA (94). A family of elF2α kinases triggered stress-induced translational arrest of mRNAs encoding “housekeeping” proteins by reducing elF2-GTP-tRNA_i_^Met^ complexes required for translation (96). The researchers found that loss of RNA m5C methylation increased angiogenin-mediated endonucleolytic cleavage of tRNA, leading to small RNA fragment accumulation from 50 tRNA molecules. Protein translation rates were reduced, and stress pathways were activated when the tRNA fragments accumulated (42). There was an increase in small tRNA fragments in response to exogenous Ang treatment. Moreover, DNMT2 overexpression protected substrate tRNAs from angiogenin-mediated degradation. Overexpression of Dnmt2 did not affect tRNAMet-ATG, a nonsubstrate of Dnmt2. Several tRNAs are methylated by Dnmt2, specifically at C38, and this methylation protects tRNAs from cleavage by the angiogenin ribonuclease (Table 2) (97).

### 4.2. Heart failure

Approximately 1% to 2% of the global adult population suffer from heart failure, which is considered an epidemic in modern times. The pathophysiological causes of heart failure encompass myocardial damage, cardiac hypertrophy, cardiac overload, cardiac electrophysiological abnormalities, and other diseases. Heart failure occurs when the heart cannot supply the peripheral tissues with sufficient blood and oxygen to meet their metabolic needs. Many studies have focused on understanding the pathophysiological mechanisms of heart failure (98). It is characterized by a reduction in genes involved in fatty acid oxidation and mitochondrial ATP production, which may be caused by insufficient energy sources. The downregulation of PGC-1*α* target genes in failing hearts is attributed, in part, to a reduction in the occupancy and recruitment of polymerase II to promoter sites, which might be a novel mechanism of metabolic perturbations in the failing heart (99). Both Dnmt2-deficient mice and Dnmt2-deficient embryonic stem (ES) cells activate RNA polymerase II (pol II), one of the key players in cardiac hypertrophy. RNA pol II activation is tightly regulated by ncRNAs such as Rn7sk and B2 RNAs, in addition to proteins such as Cdk9. The transcriptional induction of Cdk9 following an injection of small noncoding RNAs (sncRNAs) in one-cell embryos results in cardiac hypertrophy (100). In summary, as a result of decreased dissociation of the negatively regulating ncRNA component Rn7sk, the positive transcription elongation factor b (P-TEFb) complex is activated, a critical step for cardiac growth. Dnmt2 reduces cardiac growth and ES cell differentiation by phosphorylating RNA pol II, which is controlled by the ncRNA Rn7sk (Table 2) (101).

### 4.3. Cardiomyopathy

Cardiomyopathy is a condition in which the heart structure and function are affected. The term idiopathic was used historically to denote an enigmatic etiology that specifically excluded cardiovascular or systemic diseases. There are morphological subtypes of cardiomyopathy, including hypertrophic cardiomyopathy (HCM), dilated cardiomyopathy (DCM), restrictive cardiomyopathy (RCM), arrhythmogenic right ventricular cardiomyopathy (ARVC), and left ventricular noncompaction (LVNC) (102, 103). Cardiomyopathy and heart failure are prevalent presentations in mitochondrial disease resulting from inadequacies in the oxidative phosphorylation (OXPHOS) mechanism of mitochondria (104). Compared to other tissues, the muscle and heart tissue showed higher levels of m5C methylation in mice. A high methylation level and expression level of m5C-containing genes were associated with genes encoding mitochondrial and transport molecules in mouse muscle and heart tissue. In muscle and the heart tissue, the mouse VDAC1 gene contained three highly methylated sites. Generally, these data suggest that mitochondrial function may be affected by mRNA m5C modifications, particularly in organs that require the most energy, such as muscles and the heart (43). NSUN4 conditional inactivation in the heart causes respiratory chain abnormalities in germline knockouts as a result of impaired mitochondrial assembly and inhibition of mitochondrial translation. In 12S rRNA, NSUN4 increases the m5C modification at C911 residues, which regulates mitochondrial ribosome assembly and mitochondrial gene translation. Mitochondrial dysfunction occurs when Nsun4 is knocked out specifically in the heart. NSUN4 deletion in the heart caused cardiomyopathy, respiratory chain deficiency, and mitochondrial dysfunction, potentially as a result of suppressed mitoribosome assembly and mitochondrial translation (Table 2) (48). However, epigenetic modifications must be studied mechanistically to develop a more comprehensive CVD regulatory network. In the future, RNA m5C sites may prove to be an important target for assessing and treating clinical diseases. More research is needed on the molecular structures and functional pathways to fully understand the effect of RNA m5C modification and exploit it in clinical applications.

## 5. Conclusion

M5C sites are present in 34 different yeast tRNA species, but they are only found at the wobble position and position 48 of tRNA^Leu^_CAA_. Upon cellular oxidative stress, there is a dynamic change in the distribution of m5C in tRNA, with an increased distribution at the wobble position and a decreased distribution at position 48, indicating a particular response to stress (105, 106). Under stress conditions, NSUN3-mediated m5C modifications in mitochondrial tRNA^Met^ anticodon loops have been shown to decrease mitochondrial ROS production (107). Additionally, yeast, worms, and flies respond better to stress when NSUN5 is present (108). RCm1, the yeast homolog of NSUN5, catalyzes the formation of m5C2278 in 25S rRNA, which extends yeast lifespan. Specifically, Rcm1 loss alters ribosome conformation close to C2278 and translational stability, resulting in the recruitment of oxidative stress-responsive mRNAs into polysomes (109). Despite the fact that these stress responses are strongly associated with CVD, little is known about the interactions, and the detailed functions still need to be clarified.

**Figure 2:**
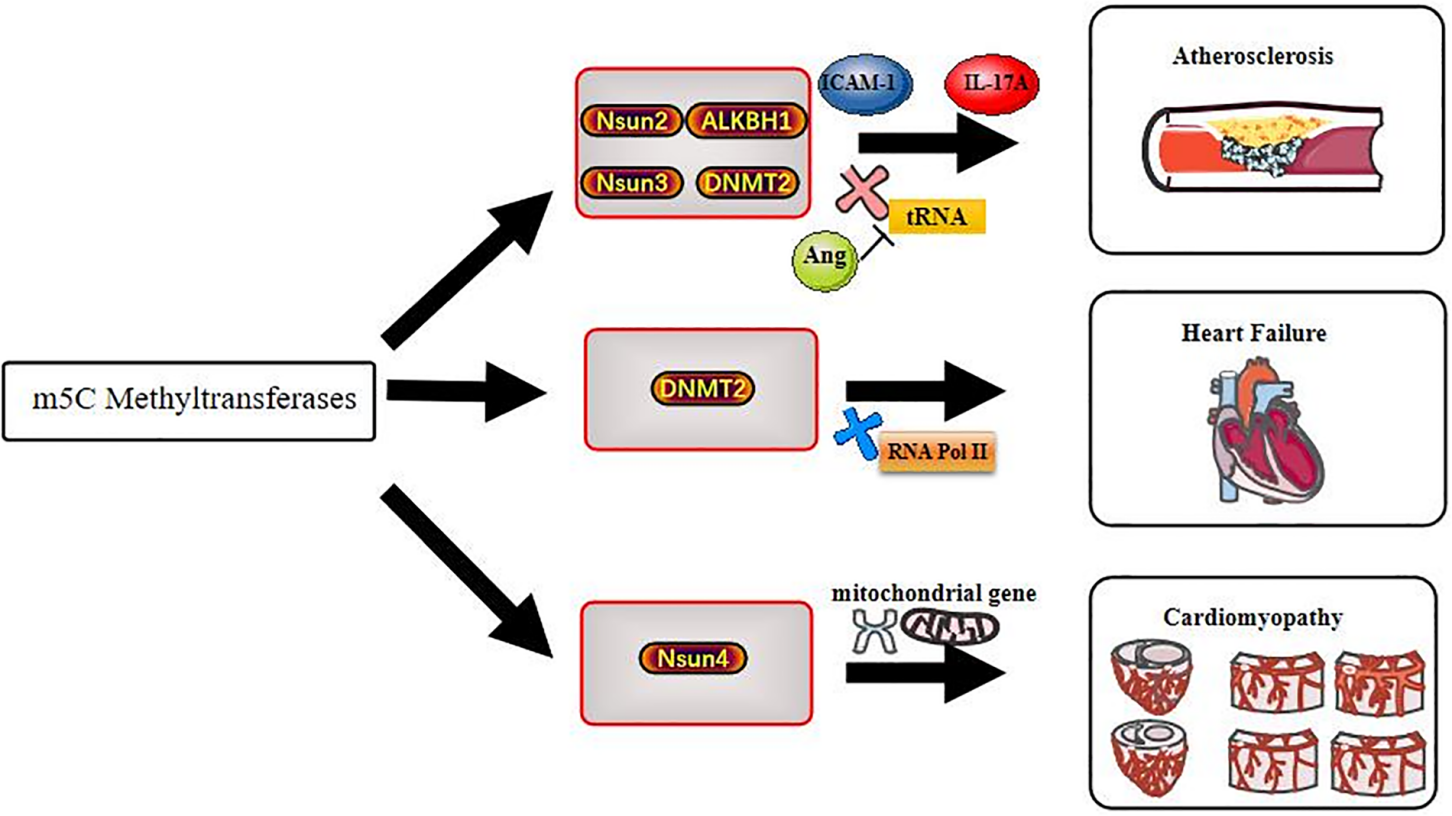
Methyltransferases act on cardiovascular diseases through m5C methylation modification.

The understanding of epigenetic modification in biological processes is increasing. In this review, we summarize the potential role of m5C methylation in CVDs and illustrate that m5C modification plays an important role in RNA epigenetics. We describe the “life cycle” of m5C methylation and its dominant function in CVDs (Figure 2). Moreover, we highlight the potential therapeutic role of interfering with m5C modifications, which could have a transformative effect on clinical medicine. Future research may uncover additional functions and underlying mechanisms of m5C methylation in CVDs, which would be important progress in the field of epigenetics.
